# Quarantine Conference

**Published:** 1889-02

**Authors:** Jerome Cochran

**Affiliations:** State Health Officer; Montgomery, Ala.


### QUARANTINE CONFERENCE.

### CIRCULAR LETTER.

This circular letter is especially addressed to the health authorities of the several States most directly interested in the protection of the South against invasions of yellow fever. Copies of it will also be sent to the governors of the several States referred to, and to the mayors of some of the more important cities for their information, and with a view of enlisting intelligent interest in the undertaking herein explained.

Under a joint resolution of our general assembly, the governor of the State of Alabama has issued to the Governors of the States of Texas, Florida, Louisiana, Mississippi, South Carolina, North Carolina, Georgia, Tennessee, Kentucky, and Illinois, invitations to appoint delegates to a quarantine conference to held in the city of Montgomery, beginning on Tuesday the 5th of March, next, and to continue for such number of days as the busines in hand may render necessary.

About two weeks ago Dr. C. P. Wilkinson, president of the board of health of the State of Louisiana, addressed a circular letter to the health authorities of these same States, suggesting a similar conference to be held in the city of Jacksonville, Florida. I have been in correspondence with Dr. Wilkinson, and .the assemblage of the proposed conference in Montgomery meets with his approval.

The object of the conference cannot be easily overrated. It is to formulate in a way that will command the confidence of the general public and of the civil and sanitary authorities of the States concerned, and in the light of our latest experience and information, the principles and regulations which should govern our Southern quarantines, and at the same time to arrange such plans for harmony and concert of action as may seem practicable and desirable.

It is earnestly desired that all of the States included in the invitation shall be represented in the conference by full delegations of such of their citizens as are best fitted to discuss the theoretical and practical problems involved in the rational administration of quarantine in the South. The occasion ought to be made a very memorial one.

The conference proper will be composed exclusively of the duly accredited delegates of the States; but other persons interested in quarantine matters will be heartily welcomed to seats on the floor, and to take such part in the discussions as under the circumstances may seem expedient.

To facilitate the work of the conference, experts, believed to be specially qualified, will be requested to formulate in advance for discussion, a series of propositions covering the subjects of maritime quarantine, railroad quarantine, municipal quarantine, depopulation of infected towns, refugee camps, panics, stampedes, disinfection, health certificates, etc.

We desire the assistance and co-operation of all who have had experience in the management of quarantines, and of all who have studied the progress of epidemics of yellow fever. Suggestions through the mails will be thankfully received.

All persons receiving this circular letter will confer a favor by acknowledging its reception, and notifying us what themselves and the communities they represent can be depended on to contribute to the success of the conference.

Address all communications to

JEROME COCHRAN, M. D.
State Health Officer.

Montgomery, Ala., January 10th, 1889.

